# Unbinding ligands from SARS-CoV-2 Mpro via umbrella sampling simulations

**DOI:** 10.1098/rsos.211480

**Published:** 2022-01-26

**Authors:** Nguyen Minh Tam, Trung Hai Nguyen, Vu Thi Ngan, Nguyen Thanh Tung, Son Tung Ngo

**Affiliations:** ^1^ Computational Chemistry Research Group, Ton Duc Thang University, Ho Chi Minh City, Vietnam; ^2^ Faculty of Applied Sciences, Ton Duc Thang University, Ho Chi Minh City, Vietnam; ^3^ Laboratory of Theoretical and Computational Biophysics, Ton Duc Thang University, Ho Chi Minh City, Vietnam; ^4^ Laboratory of Computational Chemistry and Modelling, Department of Chemistry, Quy Nhon University, Quy Nhon, Vietnam; ^5^ Institute of Materials Science, Vietnam Academy of Science and Technology, Hanoi, Vietnam; ^6^ Graduate University of Science and Technology, Vietnam Academy of Science and Technology, Hanoi, Vietnam

**Keywords:** SARS-CoV-2 Mpro, umbrella sampling, SMD, unbinding pathway, free energy

## Abstract

The umbrella sampling (US) simulation is demonstrated to be an efficient approach for determining the unbinding pathway and binding affinity to the SARS-CoV-2 Mpro of small molecule inhibitors. The accuracy of US is in the same range as the linear interaction energy (LIE) and fast pulling of ligand (FPL) methods. In detail, the correlation coefficient between US and experiments does not differ from FPL and is slightly smaller than LIE. The root mean square error of US simulations is smaller than that of LIE. Moreover, US is better than FPL and poorer than LIE in classifying SARS-CoV-2 Mpro inhibitors owing to the reciever operating characteristic–area under the curve analysis. Furthermore, the US simulations also provide detailed insights on unbinding pathways of ligands from the binding cleft of SARS-CoV-2 Mpro. The residues *Cys44*, *Thr45*, *Ser46*, *Leu141*, *Asn142*, *Gly143*, *Glu166*, *Leu167*, *Pro168*, *Ala191*, *Gln192* and *Ala193* probably play an important role in the ligand dissociation. Therefore, substitutions at these points may change the mechanism of binding of inhibitors to SARS-CoV-2 Mpro.

## 1. Introduction

SARS-CoV-2 effectuates the COVID-19 pandemic. The first human infection with severe acute respiratory syndromes was reported in Wuhan, Hubei Province, China in December 2019 [1–4]. Since then, it has caused millions of deaths worldwide. Bats have been suggested to be the carrier of SARS-CoV-2 before it jumped to and quickly transfected among humans [5]. The spreading rate is extremely high because it can be airborne [6]. In just 1 year, more than 130 million people have been infected despite international effort to contain the spread of the virus. Therefore, it is of great urgency to develop an effective therapy for community health. The approval of remdesivir as an anti-viral treatment for COVID-19 [7] may be considered as a rather controversial decision [8] because it showed poor efficacy in clinical trials [9,10]. Moreover, although two vaccines for SARS-CoV-2 have recently been approved for emergency use by the US Food and Drug Administration [11], using the vaccines to create COVID-19 herd immunity may not be possible [12]. Furthermore, especially, recent reports on new variants of SARS-CoV-2 appearing in the UK (B1.1.7) and South Africa (B1.351) can escape the neutralizing antibodies [13,14].

The main protease (Mpro) of SARS-CoV-2, a 3-chymotrypsin-like protease (3CLpro), is characterized as a pivotal enzyme associated with viral proliferation and replication [15,16]. The protease cleaves the polyproteins to polypeptides corresponding to the formation of 13 non-structural proteins (nsp) [17]. Therefore, the SARS-CoV-2 Mpro is indicated as the ultra-potent target for drug design against SARS-CoV-2 because inhibiting enzymatic activity of the protease can stop the virus from replicating.

**Figure 1.**
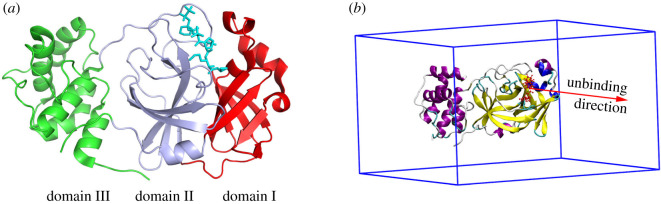
(*a*) The monomeric SARS-CoV-2 Mpro + Narlaprevir (PDB ID: 7JYC), in which, the protease was highlighted with three domains I, II and III; (*b*) starting shapes of SARS-CoV-2 Mpro + **11b** in SMD/US simulations.

The SARS-CoV-2 Mpro is a homodimer enzyme comprising of two monomers, each monomer consists of 306 residues separating into three domains I, II and III (cf figure 1*a*) [18]. In particular, the substrate binding site of the SARS-CoV-2 Mpro contains two residues, His41 and Cys145 located in the cleft formed by domains I and II [19,20]. Strong binding inhibitors to the protease normally adopt rigid contacts to these residues [21]. Furthermore, the residues *Thr26, Ser46, Asn142, Gly143, His164, Glu166* and *Gln189* were found to be crucial for the ligand-binding process [21]. Furthermore, although the two monomers interact with each other via domain II and crucially associate with the catalytic activity since the active site conformation strongly depends on the dimerization [18], the ligand-binding affinity of ligands to the Mpro *in silico* could reliably obtained from calculations performed with only the monomer instead of the dimer [22].

Computer-aided drug design (CADD) has emerged as a promising approach for screening and optimizing potential inhibitors for the biological activity of an enzyme [23–25]. In CADD, many computational approaches have been employed to estimate the binding free energy, Δ*G*_bind_, of ligands to proteins [26–48], which is associated with the experimental inhibition constant/half-maximal inhibitory concentration metrics [23,49–51]. Determination of the Δ*G*_bind_ thus is a very important issue in CADD [52,53]. In molecular dynamics (MD) simulations, a computational free energy approach is normally selected based on the balance between the computation accuracy and the consumed central processing unit resources [54–58]. Popular calculation approaches include end-to-end schemes such as the linear interaction energy (LIE) [59], molecular mechanism/Poisson-Boltzmann surface area (MM/PBSA) [34], the free energy perturbation [41], and thermodynamic integration [60] methods. These methods directly provide the binding free energy. However, the binding/unbinding pathways also provide crucial insights into the binding process of ligands to proteins. Therefore, in this work, a combination of the fast pulling of ligand (FPL) [36] and umbrella sampling (US) [61,62] simulations were employed to unbind a ligand from SARS-CoV-2 Mpro. During the simulations, the Δ*G*_bind_ value and details of unbinding pathway are characterized. In particular, the metric Δ*G*_bind_ is calculated as the difference in the potential of mean force (PMF) between *bound* and *unbound* states of the complexes. The PMF value is obtained by the weighted histogram analysis method (WHAM) [63] calculations. The unbinding pathway is able to estimate via the collective-variable free energy landscape (FEL) [64]. The representative structures of the complexes can be determined using the clustering method [65]. The obtained results revealed that the combination of FPL/US simulations was useful for accurately evaluating potential inhibitors for SARS-CoV-2 Mpro.

## 2. Material and methods

### 2.1. Structure of receptor and ligand

Twenty four three-dimensional structures of the solvated SARS-CoV-2 Mpro + inhibitor were reported in the previous work [22], in which the structure of SARS-CoV-2 Mpro was downloaded from the protein data bank with identification of 6Y2F [66] and structure of ligands downloaded from the PubChem database [67]. Details of the ligands are reported in the electronic supplementary material, table S1.

### 2.2. Atomistic simulations

GROMACS v. 5.1.5 [68] was used to carry out atomistic simulations. As mentioned above, the initial conformations of complexes (cf. figure 1*b*) were obtained from previous work [22]. Force field parameters for the protein + ions, water molecules and ligand were taken from the Amber99SB-ILDN force field [69], TIP3P water model [70] and general Amber force field [71], respectively. The MD parameters were chosen according to the previous work [21]. Particularly, the integration was carried out with a timestep of 2 fs. Cut-off for non-bonded interaction was set at a distance of 0.9 nm. The electrostatic interactions were computed using the Particle Mesh Ewald method [72], whereas the van der Waals interactions were calculated with the cut-off scheme. The solvated complexes were relaxed using energy minimization, constant number of particles, volume and temperature (NVT) and constant number of particles, volume and pressure (NPT) simulations before the steered-MD was applied.

#### 2.2.1. Fast pulling of ligand simulations

The NPT-equilibrated complex conformation was used as a starting structure of FPL calculations. During an FPL simulation the centre of mass of the ligand was pulled along the reaction coordinate, *ξ*, via an external-harmonic force. In particular, the spring constant of pulling force is *k* = 600 kJ mol^−1^ nm^−2^ and the pulling velocity is *v* = 0.005 nm ps^−1^. An FPL calculation was during an interval of 0.7 ns. The pulling parameters were referred to in the previous works [36,73]. The systemic coordinate, pulling force and pulling work were recorded every 0.1 ps.

#### 2.2.2. Umbrella sampling simulations

The optimized FPL trajectory, whereas the deviation of the pulling work to the mean is the smallest value, was used to generate US windows. The displacement of ligands between neighbouring windows is *ca* 0.1 nm. The PMF was estimated from US simulations with 21 windows and length of 2 ns per window. It should be noted that NPT simulations were performed with length of 0.1 ns to evade any staring bias. Moreover, the *C_α_* atoms were positionally restrained via a weak harmonic force to prevent the enzymic reorientation and translation during both FPL and US simulations. It helped reduce the computational effort during US simulations because we didn't have to thoroughly sample the whole protein conformational space which may be huge. The approach is reasonable because the protein conformation does not change significantly upon ligand binding.

### 2.3. Analysed tools

The two-dimensional FEL was constructed using the number of contacts within 0.45 nm between protein-ligand and ligand displacement as two reaction coordinates [64,65]. Representative structures of complexes localizing in the FEL minima were predicted using the clustering methods [65]. The WHAM [74] was used to estimate the PMF. The computed error was estimated using a bootstrapping method [75]. The Scikit-Learn library [76] was used to compute the receiver operating characteristic–area under the curve (ROC-AUC). In particular, the set of ligands were sorted and split into two groups according to their experimental binding free energy, Δ*G*_EXP_. Namely, strong binders are ligands having Δ*G*_EXP_ < −7.60 kcal mol^−1^ whereas weak binders are those with Δ*G*_EXP_ ≥ −7.60. The Python code for estimating errors using the bootstraping method and for calculating ROC-AUC is included in the electronic supplementary material.

## 3. Results

We can roughly classify proteins into two groups based on the geomorphology of their active sites. The first group includes proteins having a ‘close’ active site which requires protein structural change to allow a ligand to bind/unbind. The second one consists of proteins having an ‘open’ active site permitting its ligand to move in/out without protein conformational change. SARS-CoV-2 Mpro belongs to the second group with an ‘open’ active site that we can use pathway-based binding affinity approaches, such as FPL [36], US [62] and non-equilibrium molecular dynamics (NEMD) [39,40,77] simulations, to evaluate the ligand-binding affinity besides end-to-end free energy approaches. FPL was shown to be suitable for rapidly estimating the binding affinity of inhibitors to SARS-CoV-2 Mpro [22,58,73]. However, the obtained values cannot directly estimate the binding free energy, Δ*G*. The approach also cannot evaluate the unbinding pathway of ligands owing to the limited sampling that was generated. Δ*G* and details of the unbinding pathway can be calculated via NEMD, but the approach requires huge computing resources. US simulations, an enhanced sampling method, emerges as a potential technique to complete this task force.

**Figure 2.**
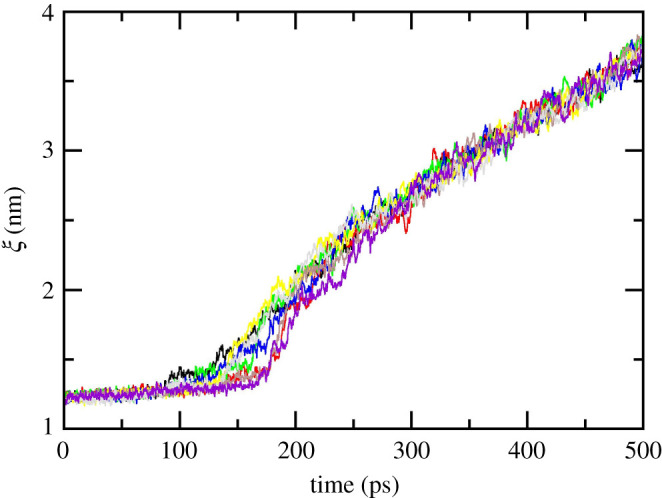
Displacement of ligand **11b** during FPL calculations.

Initial conformations used for US simulations were generated by FPL simulations with a window spacing of *ca* 0.1 nm. During FPL, the ligand displacement was recorded as shown in figure 2. Inhibitors remain in the binding cavity until the pulling force reaches a maximum value, called rupture force. Inhibitors then constantly dissociate from the binding cleft. In particular, more details about FPL results are described in the electronic supplementary material, tables S2–S4. The results obtained in this study are consistent with the recent works [21,22].

**Figure 3.**
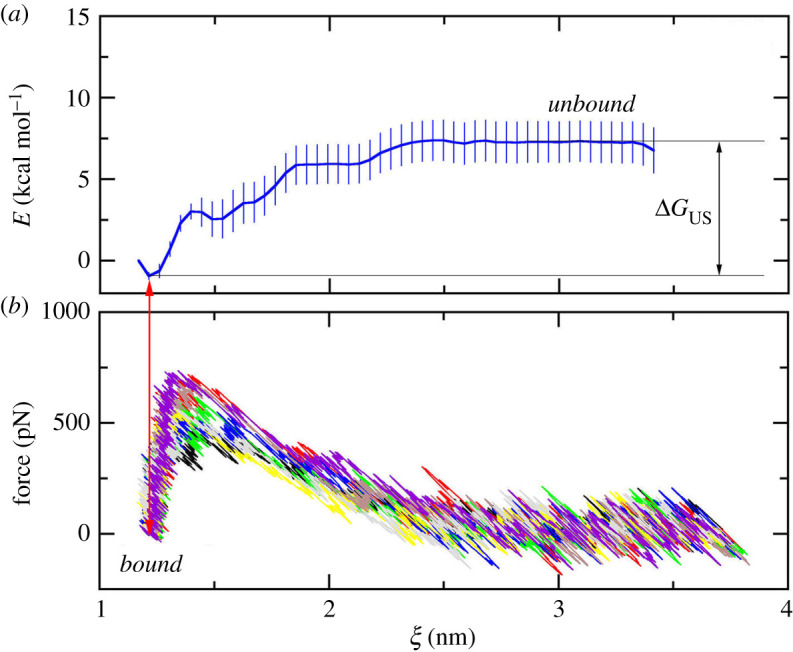
(*a*) The free energy profile via US simulations during the unbinding process of inhibitor **11b** out of SARS-CoV-2 Mpro; (*b*) pulling force via SMD simulations during the unbinding process of inhibitor **11b** out of SARS-CoV-2 Mpro.

Coordinates of solvated complexes were recorded during FPL simulations every 0.1 ps that the US windows were then extracted over the optimized FPL trajectory. The PMF values along the reaction coordinate, ξ, (cf. figure 3; electronic supplementary material, table S5) were calculated using the WHAM [74], whereas the US histograms are reported in the electronic supplementary material, table S6. The shape of the free energy profile is very consistent with previous works [48,78]. In particular, the PMF features a deep minimum at short distance and a plateau region at large distance where the non-bonded interactions between protein and ligand vanish. The observation is in good agreement with the FEL analysis as mentioned below. A free energy profile is given in figure 3 and the values of calculated binding free energy $\Delta G_{\mathrm{US}}$ are given in table 1.

**Table 1.** US binding free energy in comparison with experiments.

N^0^	name	$\Delta G_{US}$	$\Delta G_{EXP}$^a^
1	**7J**	−6.08 ± 0.61	−8.69
2	**11a**	−7.81 ± 0.91	−9.96
3	**11b**	−8.07 ± 1.29	−10.13
4	**11r**	−8.63 ± 0.78	−9.23
5	**13a**	−6.22 ± 0.58	−7.70
6	**13b**	−4.56 ± 0.66	−8.45
7	calpain inhibitor I	−4.51 ± 1.01	−6.94
8	calpain inhibitor II	−4.88 ± 0.50	−8.23
9	calpain inhibitor XII	−5.20 ± 0.70	−8.69
10	calpeptin	−5.43 ± 1.11	−6.81
11	candesartan cilexetil	−5.61 ± 0.80	−7.60
12	carmofur	−1.89 ± 1.07	−7.86
13	chloroquine	−2.94 ± 0.43	−7.41
14	dipyridamole	−5.60 ± 0.81	−8.52
15	disulfiram	−2.68 ± 0.43	−6.89
16	GC-373	−3.74 ± 0.78	−8.76
17	hydroxychloroquine	−1.80 ± 0.65	−7.58
18	MG-115	−3.14 ± 0.71	−7.53
19	MG-132	−2.57 ± 0.88	−7.41
20	narlaprevir	−2.41 ± 1.13	−6.40
21	omeprazole	−3.61 ± 1.23	−6.60
22	oxytetracycline	−7.29 ± 0.75	−7.18
23	PX-12	−2.45 ± 0.70	−6.39
24	shikonin	−2.96 ± 0.62	−6.58

^a^The experimental binding affinities were calculated from the IC_50_ value, [19,20,66,79–82] assuming that IC_50_ equals the inhibition constant *k_i_*. The unit is kcal mol^−1^.

**Figure 4.**
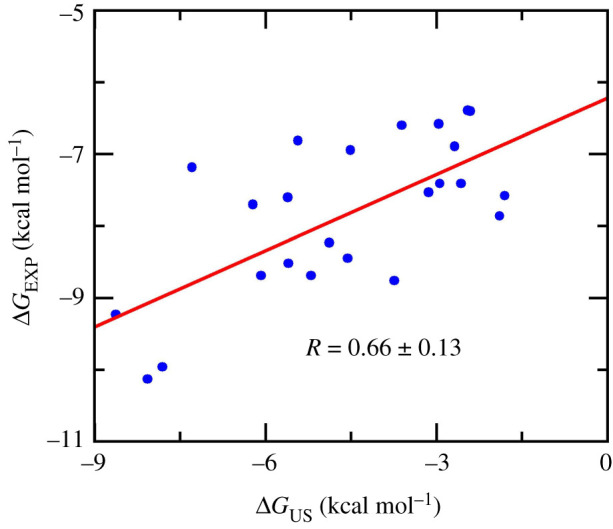
Linear correlation between $\Delta G_{\mathrm{US}}$ and $\Delta G_{\mathrm{EXP}}$. The computing error was estimated using the bootstrapping method.

The calculated binding free energy ranges from −8.63 to −1.80 kcal mol^−1^, with the mean value of $\Delta G_{\mathrm{US}}=-4.59\pm 0.41\;\mathrm{kcal}\;{\mathrm{mol}}^{-1}$. The calculated mean underestimates the experimental mean, which is of $\Delta G_{\mathrm{EXP}}=-7.81\pm 0.21\;\mathrm{kcal}\;{\mathrm{mol}}^{-1}$. The computed error bars were estimated by the standard error of the mean. The underestimation of US calculations was similar to the results when the US approach was applied to the cathepsin K (CTSK) system [62]. The inaccuracy of the force field in describing the interaction between biomolecules [83,84], which included the SARS-CoV-2 Mpro, inhibitor, water molecule, and counter ion, may cause the computed binding free energies to deviate from experimental values. The US calculations adopted an appropriate Pearson correlation with $R_{\mathrm{US}}=0.66\pm 0.13$ (figure 4), which is similar to the FPL results, $R_{W}=-0.65$, but is lower than that of the LIE, $\;R_{\mathrm{LIE}}=0.73\pm 0.09$ [21,22]. Moreover, the accuracy of computational approaches was also investigated via root mean square error (RMSE) calculation. The obtained result of US is of ${\mathrm{RMSE}}_{\mathrm{US}}=3.58\pm 0.28\;\mathrm{kcal}\;{\mathrm{mol}}^{-1}$. The ${\mathrm{RMSE}}_{\mathrm{US}}$ is slightly smaller than that from LIE calculation, ${\mathrm{RMSE}}_{\mathrm{LIE}}=4.12\pm 0.40\;\mathrm{kcal}\;{\mathrm{mol}}^{-1}$ and is significantly smaller than that from MM/PBSA, ${\mathrm{RMSE}}_{\mathrm{LIE}}=10.15\pm 1.92\;\mathrm{kcal}\;{\mathrm{mol}}^{-1}$ [21]. Furthermore, because the initial complexes obtained via the docking approach is probably far from the native structure, the US accuracy is not very high. Therefore, the systems need to relax in the MD simulations for long enough for the system to overcome local minima and reach equilibrium states [21]. The accuracy of the FPL approach was increased according to the strategy [21]. Otherwise, increasing the US window interval would probably resolve the issues since the complex in the *bound* state can reach equilibrium states. The issues should be carefully investigated in further work.

**Figure 5.**
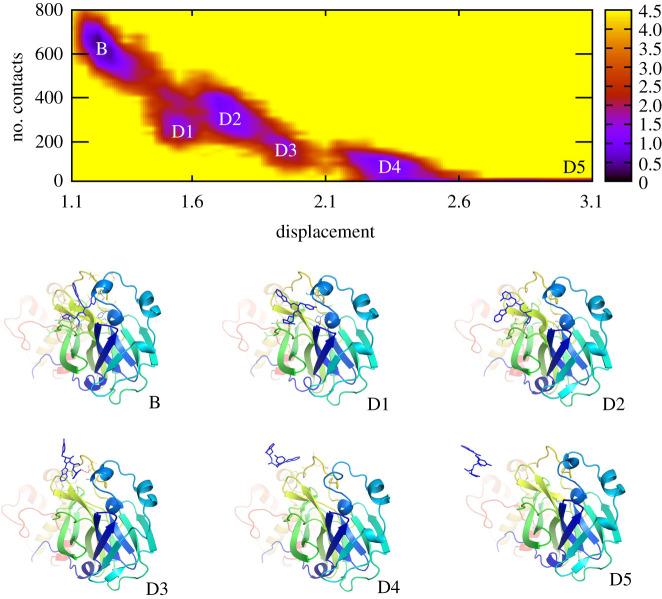
The collective-variable FEL and representative structures of SARS-CoV-2 Mpro + **11b** over US simulations.

In order to characterize the insights into the unbinding pathway of ligand dissociation, the collective-variable FEL was constructed. Two coordinates are the number of protein-ligand contacts at a cut-off distance of 0.45 nm and the ligand displacement. The two-dimensional FEL is given in figure 5 and the electronic supplementary material, table S7. Consistent with the PMF curve (figure 3*a*), the number of contacts between protein-ligand rapidly decreases when the ligand is displaced from 1.1 to 2.5 nm. Along the dissociation pathway, numerous local minima were observed. The representative structures of SARS-CoV-2 Mpro + inhibitor, which corresponded to these minima, were characterized using a data clustering algorithm with all-atom root mean square deviation chosen as pair distance and a cut-off of 0.2 nm. A representation of these conformations is described in figure 5. In particular, the SARS-CoV-2 Mpro + **11b** formed six minima, which were denoted as B, D1, D2, D3, D4 and D5. Structure B responds to the *bound* state of the complex, while structures D1–4 correspond to the transition states during the dissociated process of the ligand. Shape D5 responds to the completed dissociation state of the protein-ligand complex. Besides, the two-dimensional interaction diagram of SARS-CoV-2 Mpro + **11b** is described in the electronic supplementary material, figure S1 mentioning more details of critical residues controlling the binding process of the compound **11b**. The residues *Cys44*, *Thr45*, *Ser46*, *Leu141*, *Asn142*, *Gly143*, *Glu166*, *Leu167*, *Pro168*, *Ala191*, *Gln192* and *Ala193* probably are important elements over the ligand dissociation. These residues contribute a large number of contacts to the inhibitor over the dissociated process. The potential mutations at these points would probably alter the ligand binding pathway to SARS-CoV-2 Mpro, especially, the change in binding kinetics appeared.

In addition, ROC-AUC is commonly used to assess the predictive power of a binary classifier in separating a dataset into two classes. If we separate the set of ligands into two sets according to their experimental binding free energy as being strong binders (low binding free energy) and weak binders (high binding free energy) using some arbitrary cut-off, we can use ROC-AUC, to assess the ability of our computed free energy in distinguishing strong binders against weak binders. More specifically, if we randomly select a ligand from the strong binder set and a ligand from the weak binder set, ROC-AUC gives us the probability that the selected strong binder will have lower calculated binding free energy than the weak binder. Therefore, ROC-AUC for SARS-CoV-2 Mpro was calculated. The obtained result is $\mathrm{ROC}-{\mathrm{AUC}}_{\mathrm{US}}=0.83\pm 0.09$, indicating that US is weaker than LIE,$\;\mathrm{ROC}-{\mathrm{AUC}}_{\mathrm{LIE}}=0.86\pm 0.08$, and better than FPL, $\mathrm{ROC}-{\mathrm{AUC}}_{\mathrm{FPL}}=0.76\pm 0.11$, in classifying SARS-CoV-2 Mpro inhibitors [21].

## 4. Conclusion

We have demonstrated that the US approach provides a reliable estimate of binding free energies $\Delta G_{\mathrm{US}}$ when compared with experimental data [19,20,66,79–82]. The correlation between US binding free energy and experiments is rather high with a value of R=0.66±0.13, which is in the same range of correlation with FPL [22]. Moreover, the obtained RMSE of US simulations, ${\mathrm{RMSE}}_{\mathrm{US}}=3.58\pm 0.28\;\mathrm{kcal}\;{\mathrm{mol}}^{-1}$, is slightly smaller than that of LIE [21]. Furthermore, US is better than FPL and poorer than LIE in classifying SARS-CoV-2 Mpro inhibitors owing to ROC-AUC analysis, $\mathrm{ROC}-{\mathrm{AUC}}_{\mathrm{US}}=0.83\pm 0.09$. Clustering conformations found on local minima of the two-dimensional FEL revealed important insights into the unbinding pathway of the ligand from SARS-CoV-2 Mpro binding cavity. The obtained results imply that the residues *Cys44*, *Thr45*, *Ser46*, *Leu141*, *Asn142*, *Gly143*, *Glu166*, *Leu167*, *Pro168*, *Ala191*, *Gln192* and *Ala193* probably play an important role in the ligand dissociation. Therefore, substitutions appearing at these points possibly change the ligand binding mechanism of SARS-CoV-2 Mpro.

## Data accessibility

All relevant data and software necessary to reproduce all results in the paper are described in the material and methods of the main text and in the electronic supplementary material. Information on software is provided in the material and methods. Python code for bootstrap analysis and ROC-AUC calculations is included in the electronic supplementary material.
